# The Shan people’s health beliefs, knowledge and perceptions of dengue in Eastern Shan Special Region IV, Myanmar

**DOI:** 10.1371/journal.pntd.0007498

**Published:** 2019-06-27

**Authors:** Jian-Wei Xu, Hui Liu, Didan Ai, Yan Yu, Bian Yu

**Affiliations:** 1 Malaria Department,Yunnan Institute of Parasitic Diseases, Yunnan Provincial Centre of Malaria Research, Yunnan Provincial Key Laboratory of Vector-borne Disease Control and Research, Yunnan Provincial Collaborative Innovation Center for Public Health and Disease Prevention and Control, Pu’er City, China; 2 Institute of Pathogens and Vectors, Basic Medical College, Dali University, Xiaguang District, Dali City, China; 3 Disease Prevention Department, The Hospital of Eastern Shan Special Region IV, Mengla Township, Shan State, Myanmar; National Center for Atmospheric Research, UNITED STATES

## Abstract

Sustainable dengue intervention requires the participation of communities. Therefore, understanding the health beliefs, knowledge and perceptions of dengue among the local people can help to design locally appropriate strategies for effective interventions. A combination of qualitative semi-structured in-depth interviews (SDIs) and quantitative household questionnaire surveys (HHSs) was used to investigate the beliefs, knowledge and perceptions of dengue among the Shan people in Eastern Shan Special Region IV (ESSR4), Myanmar. The SDI was administered to 18 key informants, and the HHS was administered to 259 respondents. Only 14.7% (95% CI: 10.6–19.6%) of the HHS respondents could confirm that mosquitoes transmit dengue; 14.3% (95% CI: 10.3–19.1%) knew that piebald or Aedes mosquitoes transmit dengue; and 24.3% (95% CI: 19.2–30.0%) believed that dengue-transmitting mosquitoes mainly lived in small ponds. Merely ten (0.4%) of the 259 respondents of the HHS thought that dengue-transmitting mosquitoes bite in the day time. The people in the villages where there were outbreaks of dengue had more knowledge about dengue. This study demonstrates that the health beliefs of the Shan people were closely associated with their lifestyles, social and natural environments. To stay healthy, the Shan people clean their houses and surroundings regularly. However, their knowledge about dengue was not adequate for effective dengue control because it was mostly learned from previous dengue experiences and in a context that lacks systematic health education. Thus, in this setting, with a weak public health structure, more international support should be provided to promote the knowledge of the Shan people about dengue and to increase their sensitive awareness to dengue, which might be beneficial for social mobilization and community participation during future dengue prevention.

## Author summary

The burden of dengue has been increasing over the last five decades, and dengue fever (DF) has become one of the most rapidly spreading mosquito-borne diseases. DF has become another disease that threatens public health after malaria has become successfully controlled along the China-Myanmar border. However, it is currently not easy to contain epidemics of the dengue virus. As part of an integrated vector management approach, a community-based method is effective in the prevention of DF by tailoring the approach in a local context. Consequently, mixed methods comprising qualitative semi-structured in-depth interviews (SDIs) and quantitative household questionnaire surveys (HHSs) were used to study the health beliefs, knowledge and perceptions about dengue among the Shan people in Eastern Shan Special Region IV, Myanmar. This study found that the Shan people believed that their health was closely associated with their lifestyle and the social and physical environment in which they lived. Their beliefs originated from their primary social activities and cultural heritage. Most of their knowledge about DF was learned from previous outbreaks and interventions for the disease. The Shan people had a relatively higher level of knowledge about adult mosquito control, which they learned from previous malaria control programs, but they lacked knowledge on DF symptoms, transmission, vectors and Aedes larval breeding sites. Their knowledge about the methods of adult mosquito control could not effectively control DF. More sound health education is urgently needed to increase the local people’s knowledge of dengue and to rouse community awareness and participation in cleaning vector breeding sites. In the context of a lack of the necessary technical and financial resources, these interventions might rely more on international aid and help from neighboring countries, such as China.

## Introduction

Dengue fever (DF) is a mosquito-borne disease caused by the dengue virus. It has become a substantially increasing threat to public health and represents a challenge to health services and a burden to economies. It was recently estimated that there are 390 million dengue infections every year (95% confidence interval 284–528 million), of which 96 million (67–136 million) infections manifest clinically [1, 2]. The World Health Organization (WHO) estimated that 3.9 billion people in 128 countries are at risk for infection with dengue viruses [3] and that approximately 75% of the global population who are at risk for contracting dengue is distributed across the Asia-Pacific region [4]. However, dengue is still one of the most neglected tropical diseases, which are caused and maintained by the social and environmental determinants of health [5]. The social determinants of infectious disease control are difficult to address [6]. For that reason, the Special Programme for Tropical Diseases Research and Training from the World Health Organization has called for more research on the interplay between the demographic, social, and environmental factors in infectious disease occurrence [7]. In recent years, outbreaks of DF have occurred along the China-Myanmar-Laos border, and most of these outbreaks affected Dai or Shan ethnic communities [8, 9]. Dengue virus is primarily transmitted by *Aedes* mosquitoes, particularly *Aedes aegypti*. Water containers and discarded tires are the main productive habitats for *Aedes* [10]. The population dynamics of *Aedes aegypti* are influenced by human behavior and by the weather [11]. Outbreaks of DF may also be attributed to the social and environmental characteristics of the residents. Human behavior change communication (BCC) is one of the currently adopted strategies to reduce the *Aedes* population and dengue virus transmission [4, 11–13]. Understanding people’s health beliefs, knowledge and perception about dengue may help to generate better strategies for dengue control [14, 15]. Data about people’s health beliefs, knowledge and perceptions about dengue are still lacking at the China-Myanmar-Laos border. This kind of data may help to explore locally adaptive strategies and solutions for the next steps in dengue prevention and control. This study investigated the health beliefs, knowledge and perception of the Shan people about dengue in Eastern Shan Special Region IV (ESSR4), Myanmar. The purpose of using both qualitative and quantitative methods is to triangulate the study outcomes, to try to gain in-depth understanding and to compare any potential differences between the general community and the key informants who are supposed to have more chances to receive health information. Here, we report our findings and provide a discussion on their implications.

## Methods

### Study design

This investigation was a cross-sectional study that used mixed methods, including a quantitative HHS and qualitative SDI. Based on the number of reported dengue fever cases in 2017, three types of villages (high, low and no dengue incidence) were chosen as sample sites in Mongla Township in ESSR4, Myanmar. The SDI was administered to six key informants in each type of sampled village, so a total of 18 SDIs were conducted. The targeted sample size of the HHS, i.e., 250 household heads for the questionnaire survey, was determined based on a 95% confidence interval of the standard value normal distribution, a 5% precision and an estimated 20% of adult people who know that mosquitoes transmit dengue virus [16].

### Concept definition

Generally, people’s health beliefs are supposed to affect people’s knowledge, perception and preventive behaviors regarding diseases. In this paper, health beliefs are the general perceptions of elements related to health and diseases, not just special beliefs about dengue, i.e., people’s perception of the effect of religious, socioeconomic and natural elements on their health and their perception of disease causes. More specifically, in this article, the knowledge and perception were specifically about dengue fever, including their knowledge of the clinical symptoms, vectors, dengue virus transmission, preventive methods and their perceived risk of contracting dengue fever.

### Research tool development

First, two of the main researchers discussed the study themes, including common diseases and health problems in the community, causes of poor health, knowledge of dengue, perceived risks of dengue and preventive methods, and then drafted the guidelines for the qualitative SDI. Second, the guidelines were pretested through discussions with village leaders and health workers and were then revised and finalized according to the results of the pretests. Third, based on the qualitative SDI guidelines, a questionnaire was developed, pretested by interviewing five heads of households, and then revised and finalized. The questionnaire consisted of 55 questions that covered demographics, education, family economics, beliefs on health and disease causes, the knowledge of dengue (including symptoms, transmission, vectors and prevention), perceived risk and attitudes.

### Study site and population

ESSR4 of Myanmar adjoins the Xishuangbanna Prefecture of China [17] and has three administrative areas, i.e., Mongla Township, Nanban and Selei County. The total population of ESSR4 is approximately 110,000, and the majority of residents are Shan people (known as Dai in China, Thai in Thailand and Lao in Lao PDR), which is one of the main ethnicities in the Greater Mekong Subregion (GMS). The hot climate and adequate precipitation in ESSR4 provide a suitable environment for the growth and reproduction of mosquitoes, thereby increasing dengue transmission. Outbreaks of DF have recently occurred in Mongla Township each year [8]. The hospital of ESSR4 is the sole health facility that can perform the laboratory-based diagnosis and treatment of DF, and this hospital reported a total of 114 dengue fever cases in 2017. To compare the responses of communities with different DF experiences and to explore the potential reasons behind these responses, this study was deliberately designed to be carried out in three types of villages (V1 = no DF patients, V2 = low DF incidence, and V3 = high DF incidence). After discussion with the hospital of ESSR4, Mangjingpa and Wangnali (V1), Wangmaidao (V2) and Wangdong (V3) were selected for the study, as a common decision between us and the hospital. In 2017, V1 had 867 people and no reported DF cases; V2 had 389 people and nine reported DF cases; and V3 had 487 people and 36 reported DF cases.

### Semi-structured in-depth interviews

The qualitative data were collected by interviewing 18 key informants, with six from each type of village (i.e., V1, V2 and V3). Participants included one health worker, three village leaders and two villager representatives who were chosen by the other villagers. The interview was conducted by following the SDI guidelines. One researcher interviewed the key informants in the Shan language, and another took notes in Chinese. The contents that were discussed included local health problems, villagers’ beliefs, including religion, the causes of diseases, local dengue situations, the name for DF in the Shan ethnic language and its meaning, the linkage between mosquitoes and dengue, the time that mosquitoes bite humans, the breeding sites of dengue-transmitting mosquito larvae and people’s perceptions of the prevention of DF [18, 19].

### Household questionnaire survey

The unit of sampling of this study was a household, which was defined as all those eating from the same cooking pot. The family wealth index (FWI) was determined by the household’s physical assets, such as housing, walls, roofs, and bicycles, and was classified into five groups (Table 1). Households were selected by a simple computer randomization in V1, V2 and V3. The 50% ±10% of the targeted sample size of the HHS(250 household heads)was decided for villages with reported dengue cases and without, respectively. Chinese is the official language in the ESSR4, so the questionnaire was written in Chinese. Researchers first visited each selected household and introduced the purpose of this study, topics and related questions that would be asked. The questionnaire was subsequently administered to each household head only after oral informed consent was obtained. Investigators who understood both the Shan language and Chinese from the Hospital of ESSR4 asked every question in the Shan language and then filled out the questionnaire in Chinese [20, 21].

**Table 1:** Principal components for the construction of the family wealth index (FWI).

Family wealth index	Housing characteristics	Transportation tools	Family belongings
1 Most poor	Bamboo walls and sheet iron roofs	None	None or chickens
2	Wood walls and sheet iron roofs	Bicycles	Pigs or goats
3	Brick walls, wood girders and terracotta roofs	Motorcycles	Cattle or horses
4	Brick concrete walls and terracotta roofs	Tractors	TV sets or refrigerators
5 Least poor	Steel and concrete	Cars	Shops or elephants

### Data analysis

Records of the qualitative SDI were coded according to the contents of the questions and were then entered into cells in Microsoft Office Excel 2007. The same content records were combined by code sequencing. Two researchers independently analyzed the records of every content to generate themes. Finally, the two researchers discussed and compared their findings to finalize the findings. Quantitative data were entered in Excel 2007 and were analyzed in Epi Info 7.2. The percentage and its 95% confidence interval (CI) were calculated for each health belief, piece of knowledge and perception. A chi-squared test was used to compare the percentage of each health belief, piece of knowledge and perception across the three village categories (V1, V2 and V3) [20, 21].

### Ethics

The study was approved by the Bureau of Health of ESSR4, Myanmar. Ethical approval for this study was also granted by the Ethics Committees of the Yunnan Institute of Parasitic Diseases in China. Verbal consent was approved by the Ethics Committee as an acceptable form, as the study was interview-based and did not include any human specimens. All studied participants were adults (over 18-years-old). According to the World Medical Association Declaration of Helsinki, the purpose and procedures of the study were explained and disclosed to the participants before obtaining their consent. Participation was entirely voluntary, and participants could pass on a question, take a break or withdraw their consent from the study without providing any explanation at any time. Their consent was assumed if they did not refuse to answer questions.

## Results

### Characteristics of the respondents

The SDI was administered to 18 key informants, including nine males and nine females, ranging from 32- to 54-years-old. The questionnaire was administered to 259 household heads, and all completed questionnaires were considered valid. Among the 259 households, 211 (81.5%, 95% CI: 76.2–86%) were less poor (FWI 4–5) (Table 1). The respondents were made up of 149 females and 110 males. The mean age of the respondents was 42.3-years-old (median: 49.0, range: 18–99). A total of 225 (86.9%, 95% CI: 82.1–90.7%) respondents said that they had not received any formal health education.

### Health beliefs

Most of the participants and their family members were Buddhists. Only two of the HHS respondents were Papists, and the other three were not religious. All 18 key informants were Buddhist and agreed that, “One good turn deserves another. Evil will be recompensed with evil. The Buddha will bless and protect good people. If anyone does bad things, he or she would be punished due to their evil behaviors”. Half of 18 key informants thought that the natural environment in which they were living affected their health. In V1, three of the key informants agreed that, “In hotter weather, more people get ill”. In V2, two key informants agreed that, “Extremely hot or cold weather can make people ill”. In V3, four of the key informants agreed that, “When there are more trees, there are more mosquitoes and therefore more diseases”. Nine of 18 key informants believed that a certain link existed between household economic income and diseases. One of the key informants explained, “In poorer economic conditions, it is easier to get diseases because the poor may more easily suffer from malnutrition and be less healthy”. Fourteen of the key informants thought that clean living and working environments benefited their health. In V1, the key informants agreed that, “Poor hygiene is one of the disease causes”. In V2, the key informants agreed that, “Dirty water leads to diseases”. In V3, the key informants agreed that, “When hygiene is poor, it is easier to contract diseases” (Table 2). The results of the HHS showed that 79.9% (95% CI: 74.5–84.6%) of the respondents believed that Buddha would bless good people; 54.1% (95% CI: 48.2–60.6%) thought that anyone who did evil would be punished; 47.5% (95% CI: 41.3–53.8%) mentioned that poverty was one of the causes of disease; 62.9% (95% CI: 56.7–68.8%) believed that all natural factors, including climate, weather, water and forests, influenced human health; 87.0% (95% CI: 92.2–90.9%) believed that cleanliness and sound hygiene benefited human health; and 21.7% (95% CI: 16.7–27.2%) thought that unpolluted water was healthier than polluted water (Table 3).

**Table 2:** Thematic points of the key informant’s beliefs, knowledge and perceptions regarding dengue fever in Eastern Shan Special Region IV, Myanmar.

Themes	Village without DF (V1)	Village with low DF incidence (V2)	Village with high DF incidence (V3)
**Commonly seen diseases in communities**	Catching a cold, diarrhea, stomach diseases and tuberculosis.	Catching a cold, dengue fever, diarrhea, anemia and stomach diseases.	Dengue fever, catching a cold, febrile diseases, anemia and high blood pressure.
**Causes of ill health**			
Economic condition	Two key informants thought that diseases were related to economic conditions. The poor might suffer from malnutrition and then easily become unhealthy.	Four key informants believed that diseases were associated with economic conditions. The poor may suffer from malnutrition and be unable to go to see doctors.	Three informants believed in the existing relationship between household economic income and diseases. They said, “In poorer economic conditions, it is easier to get diseases”.
Religious/spiritual beliefs	All six key informants were Buddhists. Three of them believed that, “One good turn deserves another. The Buddha will bless and protect good people”.	All six key informants were Buddhists. Four of them agreed that, “The Buddha will bless and protect good people. Doing bad activities would be punished because of evil behaviors”.	All six key informants were Buddhists. Four of them agreed that, “One good turn deserves another. The Buddha will bless and protect good people. People who often do good things will be blessed”
Natural environment	Three key informants thought the natural environment was a determinants of health. They agreed that, “In hotter weather, more people get ill”.	Four key informants believed that the natural environment affected people’s health. They agreed that, “weather that is too hot or too cold will make people ill”.	Four key informants thought that weather changes and the environment may cause illness. They agreed that, “When there are more trees, there are more mosquitoes and therefore more diseases”.
Hygiene	Five key informants thought that keeping living and working areas clean helps health. They agreed that, “Poor hygiene is one of the causes of disease”.	Five key informants thought that it was helpful for health to keep living and working areas clean. They agreed that, “Dirty water will lead to diseases”.	All six key informants considered it helpful for health to keep living and working areas clean. They agreed that, “Poor hygiene makes it easier to get diseases”.
**Knowledge of the symptoms and pathophysiology of dengue fever (DF)**	Only one key informant mentioned fever and headache as being symptoms of DF. They did not mention any of the main symptoms such as rash, orbital pain and pantalgia.	Two key informants mentioned fever and headache as being symptoms of DF. One of them also mentioned pantalgia and joint pain.	Three key informants mentioned that high fever and pantalgia were DF clinical symptoms. One of them also mentioned rash, pantalgia and joint pain.
**Perceived risk**	Only one key informant thought that it was easy to contract DF. All six key informants had not heard about any deaths from DF. Only one key informant said, “DF may be a serious or deadly disease”. Others did not know or did not respond to the question about DF risk.	All six key informants thought that it was easy to contract DF. They had not heard about any deaths from DF. Two key informants agreed that, “DF can develop into a serious disease that may lead to death”.	All six key informants thought that it was easy to contract DF. Three of the six key informants considered DF to be a serious and deadly disease.
Transmission	In the local Shan language, DF is called “Paya Yong”, in which “Paya” is illness and “Yong” is mosquitoes, so DF is a mosquito-linked disease in the Shan language. Only two key informants thought DF was transmittable, but they thought that DF was directly transmittable from person to person through breathing, speaking or physical contact.	DF had the same name in the local ethnic language. Three key informants thought that DF was transmittable by mosquitoes. However, they also thought that DF could be directly transmitted from person to person through breathing, speaking and physical contact.	DF had the same name in the local ethnic language. Five key informants thought that DF was easily transmittable by mosquitoes, but they also thought that DF could be directly transmitted from person to person through breathing, speaking and physical contact.
**Dengue-transmitting mosquitoes**			
Type	Only one key informant mentioned that dengue-transmitting mosquitoes were piebald.	Two key informants said that dengue-transmitting mosquitoes were piebald.	Three key informants knew that dengue-transmitting mosquitoes were piebald or Aedes.
Activities and biting time	Only one key informant said that dengue-transmitting mosquitoes bite during the day and night. Another key informant said that dengue-transmitting mosquitoes bite at night. None of the key informants mentioned biting in the daytime.	Two key informants said that dengue-transmitting mosquitoes biting during the day and night. Another said that dengue-transmitting mosquitoes bite at night. None of the key informants mentioned biting in the daytime.	Three key informants said that dengue-transmitting mosquitoes bite during the day and night. Two key informants said that dengue-transmitting mosquitoes bite in the daytime. One key informant mentioned that dengue-transmitting mosquitoes bite at night.
Breeding sites	One key informant thought that dengue-transmitting mosquitoes breed in all kinds of water; two said that they bred only in small-scale pond sites. The other three key informants could not answer this question.	Two key informants thought that dengue-transmitting mosquitoes live in all kinds of water; three said that they only bred in small-scale ponds and in containers. One key informant could not respond to this question.	Two key informants thought that dengue-transmitting mosquitoes bred in all kinds of water; three said that they only bred in small-scale ponds and in containers. One key informant could not answer this question.
**Prevention**			
Environmental management and breeding site clearance	Three key informants considered cleaning the environment to be good for health. Five of the key informants thought that breeding site clearance would be able to prevent DF.	Four informants considered cleaning and hygiene to be good for health. Four of the key informants thought that breeding site clearance would be able to prevent DF.	Three key informants considered cleaning and hygiene to be good for health and were willing to participate in cleaning the community environment. Four of the informants thought that breeding site clearance would be able to prevent DF.
Protection against mosquito bites	The use of mosquito coils and bed nets were frequently mentioned as methods for preventing mosquito bites. One key informant mentioned door and window screens, and two mentioned fogging or spraying with insecticides.	The use of mosquito coils and bed nets were frequently mentioned as methods for preventing mosquito bites. Two key informants mentioned door and window screens and fogging or spraying with insecticides.	The use of mosquito coils and bed nets were frequently mentioned as methods for preventing mosquito bites. Two key informants mentioned door and window screens, and three mentioned fogging or spraying with insecticides.

**Table 3:** Results of the household questionnaire survey in Eastern Shan Special Region IV, Myanmar.

Variables	Total No. (%, 95% CI**), n = 259	No. (%, 95% CI) in V1**, n = 132	No. (%, 95% CI*) in V2**, n = 43	No. ([%, 95% CI*) in V3**, n = 84	P-value
**Demographics**					
Male household head	110 (42.5, 36.4–48.7)	52 (39.4, 31.0–48.3)	24 (55.8, 39.9–70.9)	34 (40.5, 29.9–51.7)	0.151
Age of the household head (years)					
18–45	103 (40.0, 33.8–46.0)	56 (42.4, 33.9–51.3)	16 (37.2, 23.0–27.5)	31 (36.9, 26.6–48.1)	0.6724
46–99	156 (60.0, 54.0–66.2)	76 (57.6, 48.7–66.1)	27 (62.8, 46.7–77.0)	53 (63.1, 51.9–73.4)	0.4868
**School education (years)**					
Illiterate	225 (86.9, 82.1–90.7)	113 (85.6, 78.4–91.1)	36 (83.7, 69.3–93.2)	36 (42.9, 32.1–54.1)	<0.0001
1–6	22 (8.5, 5.4–12.6)	15 (11.4, 6.5–18.0)	5 (11.6, 3.9–25.1)	2 (2.4, 0.3–8.3)	0.0503
>6	9 (3.5, 1.6–6.5)	0 (0, 0–2.8)	2 (4.7, 0.7–15.8)	4 (4.8, 1.3–11.7)	-
No response (n = 5)	5 (1.9, 0.6–4.4)	3 (2.3, 0.5–6.5)	-	2 (2.4, 0.3–8.3)	-
**Family wealth index**					
1 Most poor	2 (0.8, 0.1–2.8)	2 (1.5, 0.2–5.4)	0 (0, 0–8.2)	0 (0, 0–4.3)	-
2	5 (1.9, 0.6–4.4)	4 (3.0, 0.8–7.6)	0 (0, 0–8.2)	1 (1.2, 0–6.4)	-
3	41 (15.8, 11.6–20.9)	23 (17.4, 11.4–25.0)	6 (14.0, 5.3–27.9)	12 (14.2, 7.6–23.6)	0.7727
4	112 (43.2, 37.1–49.5)	63 (48.5, 39.7–57.3)	9 (20.9, 10.0–36.0)	40 (47.6, 36.6–58.8)	0.0054
5 Least poor	99 (38.2, 32.3–44.4)	40 (30.3, 22.6–38.9)	28 (65.1, 49.1–79.0)	31 (36.9, 26.6–48.1)	0.0002
**Social and religious beliefs**					
Poverty is a cause of ill health	124 (47.5, 41.3–53.8)	51 (38.6, 30.3–47.5)	26 (60.5, 44.4–75.0)	46 (54.8, 43.5–65.7)	0.0121
People with evil practices may be punished by diseases	141 (54.1, 48.2–60.6)	57 (43.2, 34.6–52.1)	27 (62.8, 46.7–77.0)	57 (67.9, 56.8–77.6)	0.0009
The Buddha will protect good people	207 (79.9, 74.5–84.6)	99 (75.0, 66.7–82.1)	36 (83.7, 69.3–93.2)	72 (85.7, 76.4–92.4)	0. 1264
**Natural and hygiene perceptions**					
All natural factors influence health	163 (62.9, 56.7–68.8)	62 (50.0, 38.2–55.8)	30 (69.8, 53.9–82.8)	71 (84.5, 75.0–91.5)	<0.0001
**Environments are associated with diseases?**	n = 244	n = 120	n = 42	n = 82	
Too hot	76 (31.1, 25.4–37.4)	36 (30.0, 22.0–39.0)	14 (33.3, 19.6–49.5)	26 (31.7, 21.9–42.9)	0.9143
Too cold	87 (35.7, 29.6–42.0)	46 (38.3, 29.6–47.6)	13 (30.2, 17.2–46.1)	28 (34.1, 24.0–45.4)	<0.0001
Too rainy	88 (36.1, 30.0–42.4)	24 (20.0, 13.3–28.2)	20 (47.6, 32.0–63.6)	44 (52.4, 41.2–63.4)	<0.0001
Too forested	12 (4.9, 2.6–8.4)	3 (2.5, 0.5–7.1)	6 (14.3, 5.4–28.5)	3 (3.7, 0.8–10.3)	0.0080
Rivers, streams and clear water pools near home	6 (2.5, 0.9–5.3)	2 (1.7, 0.2–5.9)	2 (4.8, 0.6–16.2)	3 (3.7, 0.8–10.3)	0.5102
Polluted water	60 (25.6, 19.3–30.5)	28 (23.3, 16.1–32.0)	9 (21.4, 10.3–36.8)	23 (28.0, 18.7–39.1)	0.6512
Poor hygiene	164 (63.3, 57.1–69.2)	69 (52.3, 43.4–61.0)	32 (74.4, 58.8–86.5)	63 (75.0, 64.4–83.8)	0.0064
**Environment benefiting health**	n = 254	n = 132	n = 41	n = 81	
Clean and sound hygiene	221 (87.0, 92.2–90.9)	108 (81.8, 74.2–89.0)	33 (80.5, 65.1–91.2)	80 (98.8, 93.3–100)	0.0007
No polluted water	55 (21.7, 16.7–27.2)	29 (22.0, 15.2–30.0)	7 (17.1, 7.2–32.1)	19 (23.5, 14.8–34.2)	0.7153
Many flowers, grass and trees around house	3 (1.2, 0.2–3.4)	2 (1.5, 0.2–5.4)	0 (0, 0–8.6)	1 (1.2, 0.03–6.7)	-
**Good hygiene can reduce diseases**	n = 256	n = 132	n = 42	n = 82	
	220 (85.9, 81.1–90.0)	105 (79.6, 71.7–86.1)	38 (90.5, 77.4–97.3)	77 (93.9, 86.3–98.0)	0.0087
**Heard about dengue**	n = 254	n = 127	n = 43	n = 84	
	85 (33.5, 27.7–39.6)	44 (33.3, 25.4–42.1)	16 (37.2, 23.0–53.3)	25 (29.8, 20.3–40.7)	0.6480
**Knowledge of DF symptoms**	n = 259	n = 132	n = 43	n = 84	
Fever	46 (17.8, 13.3–23.0)	16 (12.1, 7.1–18.9)	10 (23.3, 11.8–38.6)	20 (23.8, 15.2–34.4)	0.0532
Headache	37 (14.3, 10.3–19.1)	19 (14.4, 8.9–21.6)	5 (11.6, 3.9–25.1)	13 (15.5, 8.5–25.0)	0.8409
Orbital pain	11 (4.2, 2.1–7.5)	2 (1.5, 0.2–5.4)	1 (2.3, 0.1–12.3)	8 (9.5, 4.2–17.9)	0.0002
Pantalgia	25 (9.7, 6.3–13.9)	8 (6.1, 2.7–11.6)	6 (14.0, 5.3–27.9)	11 (13.1, 6.7–22.2)	0.1349
Rash	9 (3.5, 1.6–6.5)	3 (2.3, 0.5–6.5)	1 (2.3, 0.1–12.3)	5 (6.0, 2.0–13.3)	0.3206
Others	28 (10.8, 7.3–15.2)	15 (11.4, 6.5–18.0)	3 (7.0, 1.5–19.1)	10 (11.9, 5.8–20.8)	0.5975
Do not know or no response	87 (33.6, 27.8–39.7)	53 (40.1, 31.7–49.0)	9 (20.9, 10.0–36.0)	25 (29.8, 20.3–40.7)	0.0453
**Perceived risks**	n = 259	n = 132	n = 43	n = 84	
Easy to contract dengue	41 (15.8, 11.6–20.9)	17 (12.9, 7.7–19.8)	8 (18.6, 8.4–33.4)	16 (19.0, 11.3–29.1)	0.4140
Not easy or impossible to get DF	15 (5.8, 3.3–9.4)	12 (9.1, 4.8–15.3)	2 (4.7, 0.6–15.8)	1 (1.2, 0.03–6.5)	0.0499
A serious illness	72 (27.8, 22.4–33.7)	36 (27.3, 19.9–35.7)	13 (30.2, 17.2–46.1)	23 (27.4, 21.4–38.2)	0.9266
A deadly disease	64 (24.7, 19.6–30.4)	28 (21.2, 14.6–29.2)	12 (27.9, 15.3–43.7)	24 (28.6, 19.2–39.5)	0.4112
Do not know or no response	178 (68.7, 62.7–74.3)	89 (67.4, 58.7–75.3)	26 (60.5, 44.4–75.0)	63 (75.0, 64.4–83.8)	0.2223
Transmissibility	n = 216	n = 110	n = 36	n = 70	
Yes	54 (25.0, 19.4–31.3)	23 (20.9, 13.7–29.7)	13 (36.1, 20.8–53.8)	18 (25.7, 16.0–37.6)	0.1853
Transmittable from person to person directly	41 (20.0, 14.0–24.9)	15 (13.6, 7.8–21.5)	12 (33.3, 18.6–51.0)	14 (20.0, 11.4–31.3)	0.0315
**Knowledge of dengue causes**	n = 259	n = 132	n = 43	n = 84	
Bacteria	8 (3.1, 1.3–6.0)	4 (3.0, 0.8–7.6)	1 (2.3, 0.1–12.3)	3 (3.6, 0.7–10.1)	0.9275
Viruses	2 (0.8, 0.1–2.8)	0 (0, 0–2.8)	0 (0, 0–8.2)	2 (2.4, 0.3–8.2)	-
Mosquitoes	38 (14.7, 10.6–19.6)	10 (7.6, 3.7–13.5)	11(25.6,13.5–41.2)	17 (39.5, 25.0–55.6)	0.0032
Eat improper or dirty food	3 (1.2, 0.1–3.3)	1 (0.8, 0.02–4.1)	0 (0, 0–8.2)	2 (2.4, 0.3–8.2)	-
Animals	2 (0.8, 0.1–2.8)	1 (0.8, 0.02–4.1)	0 (0, 0–8.2)	1 (1.2, 0.03–6.4)	-
Others	10 (3.9, 1.9–7.0)	9 (6.8, 3.2–12.5)	1 (2.3, 0.1–12.3)	0 (0, 0–4.3)	-
Do not know or no response	137 (52.9, 46.6–59.1)	55 (41.7, 33.2–50.6)	22 (51.2, 35.5–66.7)	60 (71.4, 60.5–80.8)	0.0001
**Knowledge regarding dengue-transmitting mosquitoes**	n = 259	n = 132	n = 43	n = 84	
Piebald or Aedes	37 (14.3, 10.3–19.1)	13 (9.8, 5.3–16.3)	5 (11.6, 3.9–25.0)	19 (22.6, 14.2–33.0)	0.0293
Biting time	n = 259	n = 132	n = 43	n = 84	
24 hours	21 (8.1, 5.1–12.1)	8 (6.1, 2.7–11.6)	4 (9.3, 2.6–22.1)	9 (10.7, 5.0–19.8)	0.4514
Day	10 (3.9, 1.9–7.0)	2 (1.5, 0.2–5.4)	1 (2.3, 0.1–12.3)	7 (8.3, 3.4–16.4)	0.0467
Night	23 (8.9, 5.7–13.0)	13 (9.8, 5.3–16.3)	5 (11.6, 3.9–25.1)	5 (6.0, 2.0–13.3)	0.4857
Do not know or no response	205 (79.2, 73.7–83.9)	109 (82.6, 75.4–88.3)	33 (76.7, 61.4–88.2)	63 (75.0, 64.4–83.8)	0.3742
**Habitats of dengue-transmitting mosquito larva**	n = 259	n = 132	n = 43	n = 84	
All water sites	58 (22.4, 17.5–28.0)	26 (19.7, 13.3–27.5)	11 (25.6, 13.5–41.2)	21 (25.0, 16.2–35.2)	0.5678
Watered containers or small-scale ponds	63 (24.3, 19.2–30.0)	29 (22.0, 15.2–30.0)	12 (28.0, 15.3–43.7)	22 (26.2, 17.2–37.0)	0.6517
Do not know or no response	86 (33.2, 27.5–39.3)	56 (42.4, 33.9–51.3)	14 (35.6, 19.1–48.5)	16 (19.0, 11.3–29.1)	0.0018
K**nowledge regarding reducing dengue-transmitting mosquito breeding sites**	n = 259	n = 132	n = 43	n = 84	
Maintain sound hygiene	113 (43.6, 37.5–49.9)	46 (34.8, 26.8–43.6)	25 (58.1, 42.1–73.0)	42 (50.0, 38.9–61.1)	0.0100
Turn containers upside down	174 (67.2, 61.1–72.9)	93 (70.5, 61.9–78.1)	25 (58.1, 42.1–73.0)	56 (66.7, 55.5–76.6)	0.3253
Drain small-scale ponds	53 (20.5, 15.7–25.9)	34 (25.8, 18.5–30.1)	5 (11.6, 3.9–25.1)	14 (16.7, 9.4–26.4)	0.0789
Others	14 (5.4, 3.0–8.9)	8 (6.1, 2.7–11.6)	0 (0, 0–8.2)	6 (7.1, 2.7–14.9)	-
Do not know or no response	20 (7.7, 4.8–11.7)	16 (12.1, 7.1–18.9)	3 (7.0, 1.5–19.1)	1 (1.2, 0.03–6.5)	0.0133
K**nowledge regarding preventing dengue-transmitting mosquito bites**	n = 259	n = 132	n = 43	n = 84	
Door and window screens	52 (20.1, 15.4–25.5)	23 (17.4, 11.4–25.0)	12 (28.0, 15.3–43.7)	17 (20.3, 12.3–30.4)	0.0200
Use of mosquito coils	207 (79.9, 74.5–84.6)	114 (86.4, 79.3–91.7)	31 (72.1, 56.3–84.6)	62 (73.8, 63.1–82.8)	0.0300
Spraying with insecticides	72 (27.8, 22.4–33.7)	33 (25.0, 17.9–33.3)	13 (30.2, 17.2–46.1)	26 (31.0, 21.3–42.0)	0.5891
Use of bed nets	181 (69.9, 63.9–75.4)	99 (75.0, 66.7–82.1)	30 (69.8, 53.9–82.9)	52 (61.9, 50.7–72.3)	0.1235
Others	6 (2.3, 0.9–5.0)	1 (0.8, 0.02–4.1)	0 (0, 0–8.2)	5 (6.0, 2.0–13.3)	-
Do not know or no response	5 (1.9, 0.6–4.4)	1 (0.8, 0.02–4.1)	1 (2.3, 0.06–12.3)	3 (3.6, 0.7–10.1)	-
**Attitudes**					
Willing to eliminate bamboo and tree stump holes	n = 214	n = 118	n = 30	n = 66	
	161 (75.2, 68.9–80.9)	93 (78.8, 70.3–84.8)	20 (66.7, 47.2–52.8)	48 (72.7, 60.4–83.0)	0.3302
Willing to clean up dumps and turn containers upside down	n = 219	n = 124	n = 28	n = 67	
	187 (85.4, 80.0–89.8)	115 (92.7, 86.7–96.6)	23 (82.1, 63.1–93.9)	49 (73.1, 60.9–83.2)	<0.0001

**Note:** For all variables, there were a total of 259 respondents (132 in V1, 43 in V2 and 84 in V3), unless otherwise indicated.

*95% CI = 95% confidence interval

** V1 = village with no dengue fever (DF) cases in 2017; V2 = village with low DF incidence in 2017; V3 = village with a high DF incidence in 2017.

### Name for dengue fever in the Shan ethnic language and the symptoms of dengue fever

DF is named “Paya Yong” in the Shan ethnic language, in which “Paya” represents illness and “Yong” represents mosquitoes. Shan people have connected DF to mosquitoes from the literal meaning of this ethnic language phrase. However, most participants did not know the clinical symptoms of DF. Six of 18 key informants regarded fever as one of the symptoms of DF, and one of the key informants also mentioned rash, pantalgia and joint pain (Table 2). The results of the HHS showed that there was a higher proportion of respondents who did not know the DF symptoms or who did not respond to the question in V1 than in V2 and V3 (P = 0.0453); only 17.8% (95% CI: 13.3–23.0%) of the respondents mentioned fever; 14.3% (95% CI: 10.3–19.1%) mentioned headache; 4.2% (95% CI: 2.1–7.5%) mentioned orbital pain; 9.7% (95% CI: 6.3–13.9%) mentioned pantalgia; and 3.5% (95% CI: 1.6–6.5%) mentioned rash (Table 3).

### Perceived risk

Seven people perceived DF as a serious or deadly disease among the 18 key informants. In V2 and V3, all 12 key informants thought that local people could easily become infected with DF; however, in V1, only one out of the six key informants thought that local people could easily become infected with DF (Table 2). The HHS results revealed that 68.7% (95% CI: 62.7–74.3%) of the respondents either did not know about the risk or did not respond to the question regarding risk; only 27.8% (95% CI: 22.4–33.7%) of the respondents thought of DF as a serious disease; and 24.7% (95% CI 19.6–30.4%) of the respondents considered DF to be deadly (Table 3).

### Transmission and vector

Both the quantitative and qualitative data showed similar results. Ten of the 18 key informants regarded DF as a contagious disease, but they thought that DF could be transmitted from person to person directly by breathing, speaking and physical contact, etc. (Table 2). Furthermore, the HHS results revealed that 52.9% (95% CI: 46.6–59.1%) of the HHS respondents did not answer the transmission question; only 25.0% (95% CI: 19.4–31%) the respondents thought that DF was transmittable; 20.0% (95% CI: 14.0–24.9%) thought that DF could be transmitted from person to person directly; and only 14.7% (95% CI: 10.6–19.6%) of the HHS respondents confirmed that DF was transmitted by mosquitoes. Some respondents thought that bacteria, viruses, animals and improper or dirty food as one of the causes of DF (Table 3).

Six of the 18 key informants knew that dengue-transmitting mosquitoes were piebald or Aedes; they also knew that dengue-transmitting mosquitoes could bite during all 24 hours of the day. Only one key informant asserted that dengue-transmitting mosquitoes bit in the daytime, compared with four informants who thought that dengue-transmitting mosquitoes only bit at night. Thirteen key informants confirmed that dengue-transmitting larvae live in water; an additional eight of the 13 key informants confirmed that containers or small-scale pond water is the principal breeding sites for the larvae. The other five key informants did not know the breeding habitats of the mosquito larvae or they did not respond to the question (Table 2). The HHS results indicated that 79.2% (95% CI: 73.7–83.0%) of the respondents were unable to answer the vector question; only 14.3% (95% CI: 10.3–19.1%) of the respondents knew that piebald or Aedes were dengue-transmitting mosquitoes, and there were more HHS respondents who knew about dengue-transmitting mosquitoes in V2 and V3 than in V1 (P = 0.0293). Only 21 out of the 259 respondents knew that dengue-transmitting mosquitoes bit people during the day and night, and ten further confirmed that biting could occur during the daytime. More respondents knew that mosquitoes bite in the daytime in V2 and V3 than in V1 (P = 0.0467). A total of 33.2% (95% CI: 27.5–39.3%) of the HHS respondents thought that all types of water sites were the habitats of dengue-transmitting mosquitoes, and only 24.3% (95% CI: 19.2–30.0%) confirmed that the dengue-transmitting mosquito larvae were only living in containers or in the water of small ponds. More of the HHS respondents in V1 than in V2 and V3 (P = 0.0018) could not answer this question or did not respond to the habitat question (Table 3).

### Prevention

All participants showed a keen interest in this topic and thus actively discussed this issue with the investigators. Thirteen key informants knew that clearing mosquito breeding sites helps to prevent dengue transmission. Ten key informants said that good hygiene was beneficial for their health and expressed their willingness to participate in the cleaning of the community environment (Table 2). In the HHS, only 20 participants did not answer the questions regarding the control of mosquito larvae, and only five had no response to the questions. The HHS results indicated that 43.6% (95% CI: 37.5–49.9%) of the respondents knew that maintaining good hygiene was important for eliminating the habitats of the dengue-transmitting mosquito larvae; 67.2% (95% CI: 61.1–72.9%) knew that turning containers upside down was important for eliminating the habitats of the dengue-transmitting mosquito larvae; and 20.5% (95% CI 15.7–25.9%) knew that draining small-scale pond waters was important for eliminating the habitats of the dengue-transmitting mosquito larvae. More participants in V2 and V3 than in V1 understood that good hygiene helps to control DF. Regarding adult mosquito control, 20.1% (95% CI: 15.4–25.5) of the HHS respondents knew about the use of door and window screens; 79.9% (95% CI: 74.5–84.6) knew about mosquito coils; 27.8% (22.4–33.7%) knew about spraying or fogging with insecticides; and 69.9% (63.9–75.4%) knew about the use of bed nets. More HHS respondents in V2 and V3 than in V1 thought that using window and door screens (P = 0.02) and spraying with insecticides is helpful (P = 0.5891). However, more respondents in V1 than in V2 and V3 thought that using mosquito coils (P = 0.03) and bed nets (P = 0.1235) helped with dengue prevention. Additionally, more participants in V1 than in V2 and V3 (Table 3) expressed their willingness to eliminate bamboo and tree stump holes (P = 0.3302), to clean up dumps and to turn containers upside down (P<0.0001) (Table 3).

## Discussion

The dengue burden is growing and has become large enough currently. More than half of the global population lives in territories that are at risk of becoming infected with dengue [2, 4]. However, ability to contain epidemics of the dengue virus is still limited. In addition to supportive treatments, effective antiviral therapies continue to be lacking. The only licensed dengue vaccine is only partially protective [22]. Thus, intensified vector control could still be the most important strategy for DF prevention for a long time to come. Adopting protective behaviors is a multifactorial process that depends on both sociocultural and cognitive factors [23].

The Shan people have beliefs that have originated from their primary social living and culture. They believe that health is associated with all natural and social environmental factors, and that regularly cleaning their houses and surroundings to maintain sound hygiene would benefit human health. The name for DF in the Shan ethnic language (Paya Yong) connects dengue with mosquitoes, however, their knowledge and awareness of DF remain at low level. These low levels might be attributed to the fact that the local Shan people are unable to effectively benefit from the services that are provided by both the national health and education systems. Only nine of the participating household heads had completed more than six years of formal education. Thus, the participant’s low levels of knowledge and perceptions about DF might be explained by the lack of health services that they benefitted from and the inadequate formal education that they received. Meanwhile, few actions involving information, education and communication on dengue have been taken, despite the fact that the burden of DF is increasing [8], shortly after the malaria burden was effectively reduced [24]. In V2 and V3, there was a higher response rate and level of knowledge than in V1 about DF symptoms, transmission, vectors, time of day when mosquitoes bite and the breeding sites of dengue-transmitting mosquitoes. This difference might be attributed to the fact that the V2 and V3 communities have suffered from DF and thus have more experience and knowledge regarding dengue. Over half of the participants could not answer questions about the causes of DF. Only a quarter of the participants understood that dengue is transmittable, but most of the participants thought that dengue could be directly transmitted from person to person by breathing, speaking, physical contact, etc. (Tables 2 and 3). What is worse, almost all key informants did not have enough knowledge to communicate with their fellow villagers to prevent future outbreaks. Most of the participants were unsure if they were at risk of being infected by dengue. Both the qualitative and quantitative data show that the participants had difficulty with answering the questions on the risks and seriousness of DF. Only 15.8% (95% CI: 11.6–20.9%) of the participants perceived the risk of becoming infected. Furthermore, less than one-third of the participants (72/259) considered DF to be a serious disease and lethal. People in V2 and V3 were more susceptible to DF than those in V1. This indicates that their perceived risk may have correlated with their previous experience of DF outbreaks too. This Inappropriate preparedness due to the shortage of necessary resources might be one of the reasons for the outbreaks of DF in ESSR4, Myanmar.

More of the participants in V1 than in V2 and V3 showed a willingness to eliminate bamboo and tree stump holes, to clean up dumps and to turn containers upside down. Based on some of the SDI data, local governments have frequently required villagers to clean their houses, public spaces in the community and the surrounding environment during DF outbreaks. In response to DF outbreaks, three ten-person teams in V3 would alternate to conduct spraying and fogging with insecticide once a week. During our communications, we felt that people in V2 and V3 became bored while hearing about these requirements. One villager said, “The campaign just stopped a few days ago”. This ennui might lower their willingness to perform environmental management and may become a new challenge for the sustainability of intensive vector control.

Because of the malaria control program, the residents’ knowledge of adult mosquito control was improved to a certain level across ESSR4 [24]. In the HHS, 79.9% (95% CI: 74.5–84.6%) and 69.9% (95% CI: 63.9–75.4%) of the participants mentioned that they had used coils and bed nets, respectively. More of the participants in V1 than in V2 and V3 knew how to use mosquito coils and bed nets. However, only a few people knew that dengue-transmitting mosquitoes bite during the day and that small-scale pond water is the main breeding site of dengue-transmitting mosquito larvae. Most participants knew to protect themselves from the bites of adult mosquitoes, but they did not know that eliminating the habitats of dengue-transmitting mosquito larva is beneficial for the control of dengue vectors (Tables 2 and 3). Combining effective interventions against multiple arboviral diseases has been suggested by some scholars as one of the most cost-effective and sustainable strategies for the reduction of vector-borne diseases [25, 26]. However, another study that conducted a meta-analysis documented that indoor residual spraying (IRS) did not significantly impact DF infection risk [27]. Both this literature and our study results illustrate that knowledge regarding the control of adult mosquitoes is not sufficient for controlling the dengue vector.

Unavoidably, this study was constrained by two obvious limitations. First, one of the objectives of the qualitative SDI was to triangulate the study outcomes of the quantitative methods. Much more attention was paid to this objective in the qualitative SDI, and not enough nuanced data were collected. This limited our ability to delineate the outcome differences between the qualitative SDI and the quantitative HHS despite the qualitative and quantitative outcomes also showed that the key informants had better knowledge than overall community people. Further qualitative study should been needed. Second, the response rates of the HHS participants to certain questions were not high enough, and thereby might have caused information bias. However, all investigators felt that the Shan People were very friendly, and we very much enjoyed collaborating with them during the field research. This means that the Shan people may have been unlikely to reject answering questions that they could answer. When the individuals had no response to a specific question it was mainly because they did not know the answer. To add to this disclosure, we combined the answers of “I don’t know” and no response into a single category during analysis.

In conclusion, the Shan people believe that health is associated with all natural and social environmental factors, and that regularly cleaning their houses and surroundings to maintain sound hygiene would benefit human health. However, their knowledge and aware sensitivity of DF remains at low level, and most of their knowledge and awareness was learned from previous experiences in controlling malaria and dengue. Thus, in this setting with a weak public health structure, more international support should be provided to promote the knowledge of the Shan people about dengue and to increase their aware sensitivity to dengue. With proper guidance, social mobilization and community participation might help increase the perception of DF and the involvement of the Shan people for dengue control.
